# Avenanthramide-C as Alzheimer’s Disease-Modifying Therapy: Early and Sustained Intervention Prevents Disease Progression in Mouse Models

**DOI:** 10.3390/cells14110826

**Published:** 2025-06-02

**Authors:** Alen Benhur Pravin Nathan, Areeba Aziz, Semyeong Choi, Seunghee Lee, Seyoung Jeon, Hyung-Seok Kim, Jonghyun Cho, Jihoon Jo

**Affiliations:** 1 Department of Biomedical Sciences, Chonnam National University Medical School, Gwangju 61469, Republic of Korea; alenbenhurphd@gmail.com (A.B.P.N.); azizareeba5@gmail.com (A.A.);; 2 Department of Medicinal Biotechnology, College of Health Sciences, Dong-A University, Busan 49236, Republic of Korea; semyeongchoi@gmail.com; 3 Department of Forensic Medicine, Chonnam National University Medical School, Gwangju 58128, Republic of Korea; veritas@jnu.ac.kr

**Keywords:** AD, Avn-C, LTP, Aβ1-42, tau hyperphosphorylation, neuroinflammation, Microglia

## Abstract

Most approved drugs for Alzheimer’s disease (AD) are indicated for early to moderate stages and primarily target amyloid-beta or neurotransmitter systems. While these treatments may slow cognitive decline, they do not halt disease progression and are often limited by high cost and modest efficacy. Natural compounds are increasingly being explored as alternative interventions. Our previous study showed that oral administration of Avenanthramide-C (Avn-C), a natural polyphenol from oats, for 14 days from early AD stages improved cognition and reduced neuroinflammation in AD mice. To assess its long-term potential, in this study we extended Avn-C treatment to three months starting from early disease stages in 5xFAD and Tg2576 models. Sustained administration preserved recovered long-term potentiation (LTP) by maintaining AMPK activation and inhibiting caspase-3 and GSK3β, thereby reducing amyloid accumulation and tau hyperphosphorylation in the hippocampus. Avn-C also maintained anti-inflammatory effects by suppressing NF-κB-mediated proinflammatory cytokine release and preventing chronic microglial activation. This promoted microglial coverage of plaques in vivo and enhanced phagocytosis in vitro. Our findings suggest that early and sustained Avn-C treatment preserves cognitive function, modulates multiple pathological pathways, and may slow or prevent AD progression by targeting early neurodegenerative processes before irreversible damage occurs.

## 1. Introduction

Alzheimer’s disease (AD) is a progressive brain disorder that leads to memory loss, cognitive decline, and behavioural changes due to increasing brain cell degeneration [1,2]. Researchers have discovered that the progression of neuronal degeneration in AD is likely due to several factors, mainly a gradual increase in abnormal amyloid and tau protein processing and accumulation, mitochondrial dysfunction, and neuroinflammation [3,4,5]. Neuroinflammation is now recognized as a key contributor to AD and memory loss. While microglia do not initiate AD, human brain imaging studies show that they shift from a protective to a damaging state during disease progression, releasing proinflammatory cytokines that disrupt synapses [6,7]. An early event in AD is impaired synaptic plasticity and dysfunction rather than neuronal loss shown in AD transgenic mice [8]; safeguarding the synapses while modifying and preventing the disease pathology at the early stage may contribute to halting or slowing the rate of cognitive decline.

Current AD drug development shows that 28% of clinical trials focus on amyloid and tau, while the remaining 72% target other pathways, especially neuroinflammation and synaptic plasticity [9]. Currently, available drugs show modest benefits, although their place in AD treatment is highly debated. There are three FDA-approved cholinesterase inhibitors available to treat symptoms from mild to moderate disease stages: galantamine, rivastigmine, and donepezil, which help to increase the level of acetylcholine essential for learning and memory. While this medication offers symptom relief, they are not known to stop the progression of AD [10,11]. Interestingly, FDA-approved drugs Lecanemab and Donanemab have shown promise in slowing AD progression in early-stage patients by targeting brain amyloid proteins, helping delay plaque buildup and cognitive decline [12]. Even though the FDA-approved drugs are designed to improve neuroprotection from the early AD stage, the drug development pipeline is criticized for its narrow targeting therapies, which have shown limited long-term efficacy [13]. In recent decades, significant progress has been made in understanding the molecular and cellular mechanisms driving AD pathology and cognitive decline. Key signaling molecules such as AMP-activated protein kinase (AMPK) and Glycogen synthase kinase 3β (GSK3β) are central to major AD features like amyloid-β metabolism and tau hyperphosphorylation. Notably, several pharmacological AMPK activators have shown promise in enhancing memory function [14,15,16], specifically lowering Aβ metabolism and caspase-3 mediated apoptosis [17,18,19]. Metformin, Tideglusib, and several natural compounds like quercetin, oxyphylla A, icariin, and isoorientin have been shown to activate AMPK and inhibit GSK3β, offering neuroprotection in AD. However, studies have highlighted limitations, such as toxicity, poor bioavailability, difficulty crossing the blood–brain barrier (BBB), and potential impairment of cognition with long-term use [20,21]. Reasonable drug treatment expectations include preventing disease progression at the early disease stage with long-term efficacy, multi-target mechanisms, stabilization, or slower than the expected rate of cognitive decline.

Our earlier studies demonstrated the anti-Alzheimer’s potential of Avenanthramide-C (Avn-C), a natural oat-derived compound. When given orally at 6 mg/kg for 14 days from the early disease stage in AD mice, Avn-C crossed the blood–brain barrier, restored long-term potentiation (LTP), and reduced neuroinflammation and apoptosis in the hippocampus [22,23,24]. Building on this, the current pre-clinical study assessed its long-term impact in halting disease progression. The results provide a strong foundation for developing Avn-C as a future therapeutic option for early-stage Alzheimer’s disease.

## 2. Materials and Methods

### 2.1. Mouse Models

Wild-type (WT) (C57BL/6 J, 9–10 months old), 5xFAD (*APP KM670/671NL* (Swedish), *APP V717I* (London), *APP I716V* (Florida), *PSEN1 M146L*, *PSEN1 L286V*, 4–5 months old), Tg2576 (*APP KM670/671NL*, 6–7 months old; Jakson Laboratory, Bar Harbor, ME, USA) male mice were used. All experimental animals were housed in individually ventilated cages with ad libitum access to food and water, under a 12 h light/dark cycle, and maintained at a 22–30 °C temperature. The sample size (*n*) indicates the number of animals used for each experiment.

### 2.2. Hippocampal Slice Preparation

The experiment was conducted between 9:30 a.m. and 10:00 a.m. The animals were sacrificed by cervical dislocation, and the brain was quickly removed and transferred to ice-cold artificial cerebrospinal fluid (aCSF; 124 mM NaCl, 3 mM KCl, 26 mM NaHCO_3_, 1.25 mM NaH_2_PO_4_, 2 mM CaCl_2_, 1 mM MgSO_4_, and 10 mM glucose). A midsagittal cut was made in the brain, and one hemisphere was returned to ice-cold aCSF until required. The hippocampal slice was prepared by transverse cut (400 µm thick) using a Mcilwain tissue chopper (Mickle Laboratory Engineering Co. Ltd., Dorking, Surrey, UK), and the slices were kept for recovery for one hour in aCSF, constantly perfused with a gas mixture of 95% O_2_ and 5% CO_2_ at room temperature to reduce swelling and damage in the superficial layer of the slice to obtain stable response for LTP recording.

### 2.3. Electrophysiology

Electrophysiology experiments were conducted after the recovery for approximately 60 min from the slice preparation procedure. The stimulation electrode was placed in the subiculum and CA3 (Schaffer Collateral pathway). Extracellular field potentials were recorded in the CA1 region using the microcapillary electrodes (3–5 MΩ) filled with 3M NaCl. The Nickel80/Chromium20 (o.d. 0.066 mm) bipolar two electrodes were used to deliver stimuli alternatively (each electrode 0.013 Hz) in the stimulation electrode. After establishing the stable baseline for 30 min, LTP was evoked by two trains (2X) of high-frequency tetanus stimuli (each 100 Hz, 1 s: repeated after a 30 s interval), field excitatory postsynaptic potentiation (fEPSP) amplitude was recorded for at least 60 min, and the slope of the evoked fEPSP response was measured and expressed relative to the normalized preconditioning baseline. Data were collected by the NI USB-6251 data acquisition module (National Instruments Corp., Austin, TX, USA), amplified by an Axopatch 200B amplifier (Axon Instruments, Union City, CA, USA), and captured and analyzed by WinLTP (version 2.32) software.

### 2.4. Avn-C Preparation and Administration

The natural oat extract Avenanthramide-C (Avn-C) was obtained in a previously established, extracted, and purified form, as described in our earlier study [23]. Avn-C was dissolved in 10% Kolliphor (non-ionic surfactant, solubilizing agent made from polyethoxylated castor oil, commonly used in preclinical and clinical research to solubilize hydrophobic drugs) to a final 1 mg/mL concentration, incubated at 37 °C for 15 min, and ultrasonicated for 10 min. Early-stage AD mice (5xFAD and Tg2576) were divided into vehicle and Avn-C-treated (6 mg/kg/day, oral) groups for each model. Avn-C was orally administered daily at ~4:00 p.m. for three months. Vehicle groups received an equivalent volume of Kolliphor in distilled water without Avn-C.

### 2.5. Western Blot

The hippocampal tissue lysates were prepared with ice-cold RIPA buffer (AKR-190; Cell Biolabs, San Diego, CA, USA) together with the protease inhibitor cocktail (210205; Cell Biolabs) and homogenized with a pestle on ice. The hippocampal tissue protein extract was quantified by the BSA assay kit (Thero Scientific, Waltham, MA, USA). The protein samples (40 µg) were separated in 6–18% SDS-polyacrylamide gels and transferred to the polyvinylidene difluoride (PVDF) membrane. After blocking the membrane with 1 × Rapid Block solution (Amresco, distributed by VWR, Radnor, PA, USA) for 10–15 min, the blots were incubated for overnight at 4 °C with primary antibodies (at 1:1000) specific for anti-phospho-AMPK⍺ (Thr172) Rabbit, anti-Cleaved Caspase-3 Rabbit, anti-β-Amyloid42 Rabbit, anti- phospho- IKK⍺/β (Ser176/180) Rabbit, anti-phosphor-IKB⍺ (Ser 32/36) Mouse, anti-phospho-NF-kB (p65) (Ser536) Mouse, anti-TNF⍺ Rabbit, anti-IL6 Rabbit, anti-IL1β Mouse, anti-phospho-GSK3β (Ser9) Rabbit, anti-phospho-TAU (Ser396) mouse, anti-phospho-TAU (Ser404) Rabbit, anti-phospho-TAU (Ser202) Rabbit, anti-phospho-TAU (Thr231) Rabbit, anti-Iba1 rabbit, anti-β-Actin Rabbit (Cell Signaling, Danvers, MA, USA), Anti-Amyloid Precursor Protein (APP) Mouse (Abcam, Cambridge, MA, USA), and anti-BACE1, Mouse (Santa Cruz Biotechnology, Inc., Dallas, TX, USA). After three 10 min washes, membranes were incubated with rabbit or mouse IgG secondary antibodies (1:5000, Cell Signaling Technology) in 1 × TBST for 2 h at room temperature. Protein bands were visualized using an enhanced chemiluminescence detection system (Millipore, Burlington, MA, USA), and band intensities were quantified using FIJI software (NIH, Bethesda, MD, USA). All values were normalized to β-actin levels.

### 2.6. Immunohistochemistry (IHC)

The hippocampus was fixed in 4% paraformaldehyde, cryoprotected in 15% and then 30% sucrose overnight, and embedded in FSC 22 Clear OCT compound (Surgipath Medical Industries Inc., Richmond, IL, USA) for 1 h at room temperature. Tissue sections were then cut at 50 µm thickness using a Leica CM1860 cryostat. Brain sections were mounted onto glass slides and permeabilized with 0.5% Triton X-100 for one hour. After washing with 1x PBS, the sections were blocked with a buffer containing 1% BSA, 10% Goat serum, and 0.5% Triton X-100. Sections were incubated with primary antibodies, anti-Iba1 (1:1000), anti-β-Amyloid42 Rabbit (D54D2), anti-β-Amyloid42 Mouse (D3D2N), and anti-phospho-TAU (Ser404) (Rabbit, Cell Signaling, Danvers, MA, USA), overnight in 4 °C. After being washed with 1x PBS, sections were probed with Anti-Rabbit IgG (H+L), F (ab’)2 Fragment (Alexa Fluor 488 conjugated) and Anti-mouse IgG (H+L), F (ab’)2 Fragment (Alexa Fluor 555 conjugated) (Cell Signaling, Danvers, MA, USA) secondary antibodies (1:500) at room temperature for two hours, followed by washing with 1xPBS three times for 10 min each. Slides were mounted with ProLong Gold Antifade reagent with DAPI (Cell Signaling, Danvers, MA, USA). Confocal Z-stack images of the hippocampal slices were obtained from a Zeiss LSM 510 confocal laser scanning microscope (Carl Zeiss). The FIJI software was used to analyze the images.

### 2.7. Amyloid-β (1-42) Preparation

Aβ (1-42) peptide (Abcam, Cambridge, UK) was prepared according to the manufacturer’s protocol for in vitro studies. Lyophilized peptide was equilibrated at room temperature for 15 min, then monomerized by dissolving to 1 M in 100% HFIP and dried under nitrogen gas. To form oligomers, the peptide was resuspended in DMSO (5 mM), sonicated in a water bath for 10 min, diluted to 1.0 µM in serum-free DMEM-F12, and incubated at 4 °C for 24 h.

### 2.8. BV-2 Cell Culture

The immortalized murine BV-2 microglial cells were cultured in Dulbecco’s Modified Eagle Medium (DMEM)-F12 (D6421, Sigma-Aldrich, St. Louis, MO, USA) supplemented with 5% FBS (Wel Gene) and 100 units/mL Antibiotic Antimycotic solution (Sigma-Aldrich). The cells were maintained at 37 °C in a humidified atmosphere containing 5% CO_2_ and 95% air. They were passaged every three days when they reached approximately 75% confluence.

### 2.9. Phagocytosis Assay

BV-2 microglial cells (1 × 10^5^/well) were seeded on Poly-D-Lysine (Gibco, Grand Island, NY, USA)-coated 22 mm coverslips in 6-well plates and incubated overnight in a sterile cell culture incubator. The next day, cells were treated with 1.0 µM Aβ1-42 oligomers in serum-free DMEM-F12 for 30 min to induce activation. Groups were divided by time points (3, 6, and 12 h), with or without 50 µM Avn-C treatment. At each time point, phagocytic activity was assessed by incubating cells with crimson fluorescent microspheres (Thermo Fisher Scientific, Waltham, MA, USA, 1 µm) coated with BSA, 50 beads/cell at 37 °C for 30 min. Cells were washed and processed for immunocytochemistry.

### 2.10. Immunocytochemistry

BV2 microglial cells from the phagocytosis assay were washed three times with 1x HBSS (5 min each) to remove non-internalized microspheres, then fixed with 4% paraformaldehyde. After another HBSS wash, cells were permeabilized with 0.5% Triton X-100 for 10 min and blocked with 5% BSA for 1 h at room temperature. Cells were incubated overnight at 4 °C with anti-Iba1 primary antibody (1:1000, Cell Signaling), followed by PBS washes and incubation with Alexa Fluor 488-conjugated secondary antibody (Anti-Rabbit IgG F (ab’)2, Cell Signaling). After washing, coverslips were mounted using ProLong Gold Antifade reagent with DAPI (Cell Signaling, Danvers, MA, USA). Imaging was performed using a Zeiss LSM 510 confocal microscope (Carl Zeiss, Jena, Germany) and analyzed with FIJI software.

### 2.11. Statistical Analysis

All data are means ± S.E.M. analyzed, and graphs were made using Microsoft Excel software. Statistical analyses were performed using Prism (GraphPad), the unpaired *t*-test (two-tailed), and one-way ANOVA with post hoc Tukey’s test. Values of *p* > 0.05 were considered not significant (n.s.); values of * *p* < 0.05, ** *p* < 0.01, and *** *p* < 0.001 were deemed significant.

## 3. Results

### 3.1. Long-Term Avn-C Administration from the Early AD Stage Restores and Maintains LTP While Preventing Further Synaptic Impairment

**Figure 1:**
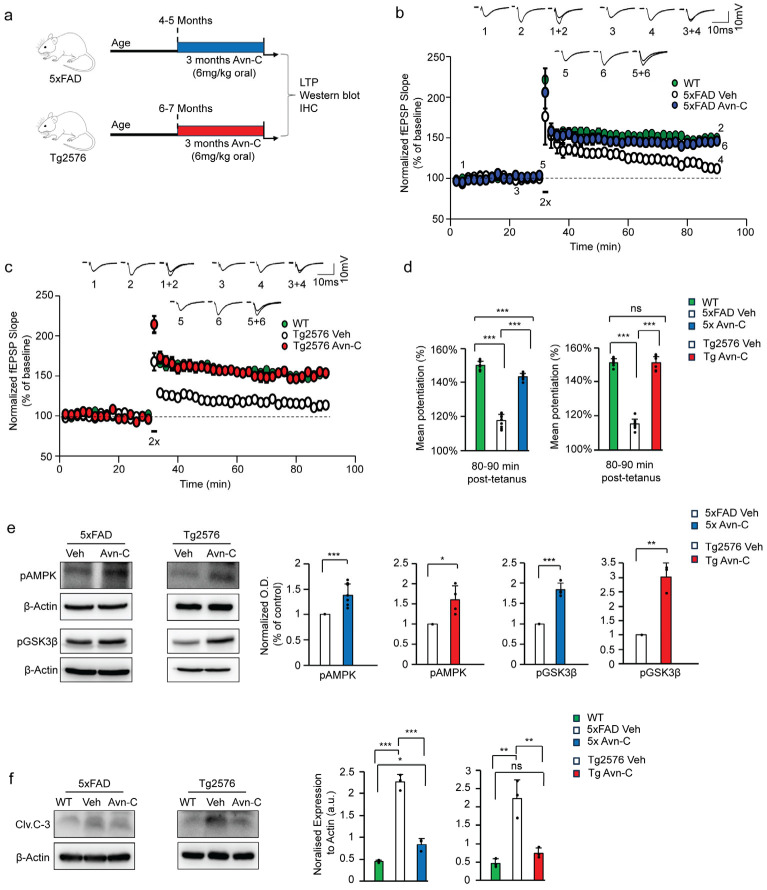
Long-term Avn-C administration from the early AD stage preserves LTP and active AMPK levels while inhibiting the activation of GSK3β and caspase-3 in AD mouse models. (**a**) Schematic representation of long-term (three months) Avn-C oral administration initiated at the early AD stage in 5xFAD and Tg2576 AD model mice and further experiments. (**b**,**c**) fEPSP recording from WT, 5xFAD, and Tg2576 (vehicle and 3 months Avn-C) AD mice showing that Avn-C administration restored and maintained LTP compared to the vehicle, recorded for one hour after 30 min stable baseline maintenance from the CA3 region of the hippocampal slice. The LTP was evoked by the two trains (2x) of tetanus stimulation at 100 Hz for 1 s. The black box symbol in the LTP data indicates tetanus stimulation (2X). (**d**) The level of mean potentiation of the last 10 min (80–90 min) post-2x tetanus shows the significant effect of Avn-C in maintaining the synaptic potential (WT-149% ± 2%, 5xFAD vehicle-111% ± 2%, 5xFAD Avn-C 145% ± 3%) (*n* = 5), (WT-151% ± 0.7%, Tg2576 vehicle-115% ± 4%, Tg2576 Avn-C-151% ± 2%) (*n* = 6). (**e**) Western blot and quantitative validation of long-term Avn-C oral administration starting from early AD stage, 5xFAD, and Tg2576 (vehicle and 3 months Avn-C treated) AD mice hippocampal lysate shows active phosphor-AMPK Thr172 (*n* = 4) and inhibited GSK3β by phosphorylation at ser9 (S9GSK3β,) 5xFAD (*n* = 8) and Tg2576 (*n* = 3). (**f**) During AD progression, untreated vehicle 5xFAD and Tg2576 show elevated Cleaved caspase-3 levels compared to WT mice; Avn-C administration maintained the lower Cleaved caspase-3 levels in both AD model mice (*n* = 3) and prevented further activation. Mean ± S.E.M.s, overall differences among groups were analyzed using unpaired two-tailed *t*-test, one-way ANOVA (*** *p* < 0.001) and pairwise comparisons between groups using post hoc Tukey’s test. Statistical significance is indicated as *** *p* < 0.001, ** *p* < 0.01, * *p* < 0.05, and ^ns^ *p* > 0.05 (no significance).

Based on our previous findings, oral administration of Avn-C at 2 and 4 mg/kg in AD mice at the early disease stage did not yield significant effects on synaptic plasticity. However, a 6 mg/kg dose resulted in a significant restoration of long-term potentiation (LTP) [22]. Therefore, in the present study, we selected 6 mg/kg as the effective dose and administered it orally from the early stage of Alzheimer’s disease. Treatment was continued for three months during disease progression in two AD mouse models: 5xFAD and Tg2576. This approach assessed whether Avn-C could modify the disease course by maintaining synaptic function and preventing further pathological progression. Following the treatment period, hippocampal tissues were harvested for downstream biochemical and electrophysiological analyses (Figure 1a). Long-term potentiation (LTP), a key mechanism of synaptic plasticity, offers essential insights into memory and cognitive deficits. In Alzheimer’s disease models like 5XFAD and Tg2576 mice, LTP is notably impaired [25] and also represents AD pathology almost similar to that observed in human patients, which helps vital research to test the development of new therapeutic drugs for preventing severe cognitive impairment [26,27]. For assessing the LTP, the amplitude of field excitatory postsynaptic potentials (fEPSP) in the CA3-CA1 (Cornu Ammonis) pathway from acute brain hippocampal slices of wild-type (WT), vehicle, and Avn-C-treated 5xFAD, Tg2576 mice were measured. After 30 min of fEPSP baseline maintenance (0–30 min), the LTP was induced by 2x tetanus induction (each 100 Hz, 1 s; repeated after a 30 s interval) and recorded for 1 h (30–90 min) from the stratum radiatum of the CA1 region to find the synaptic strength and plasticity. LTP was markedly suppressed in untreated 5xFAD and Tg2576 mice, with significantly reduced fEPSP responses compared to age-matched wild-type vehicles. However, three months of oral Avn-C treatment from the early disease stage restored and sustained LTP in both AD models, as evidenced by increased fEPSP amplitudes relative to untreated vehicles (Figure 1b,c).

Additionally, we analyzed the mean potentiation during the final 10 min (80–90 min) following 2× tetanus stimulation. The fEPSP results indicated that AD progression severely impaired synaptic function, with both untreated AD models failing to maintain synaptic potentiation compared to wild-type mice (WT-149 ± 2%, 5xFAD Con-117 ± 3%, *p* < 0.001), (WT-151 ± 2%, Tg2576 Con-115 ± 2%, *p* < 0.001). Interestingly, early oral administration of Avn-C significantly restored synaptic potentiation, and continued treatment over three months sustained this recovery, effectively preventing further synaptic impairment in both AD mouse models (5xFAD 3 months Avn-C-143 ± 1%; *p* < 0.001, compared to 5xFAD vehicle) (Tg2576 3 months Avn-C-151 ± 3%; *p* < 0.001, compared to Tg2576 vehicle). Moreover, during the final 10 min of fEPSP mean potentiation, compared to the WT mice, the 5xFAD group treated with Avn-C showed only a ~4% difference (*p* < 0.001), indicating a near-complete recovery of synaptic function, and the Tg2576 mice treated with Avn-C showed no significant difference from wild-type (*p* > 0.05), suggesting that Avn-C had already restored synaptic potentiation to near-normal WT levels (Figure 1d). Overall, long-term Avn-C administration in 5xFAD and Tg2576 mice effectively restored and maintained LTP, with levels comparable to wild-type vehicles (ANOVA, *p* < 0.001, *n* = 6), in contrast to untreated AD mice. These findings highlight that sustained oral Avn-C treatment, initiated at the early stage of Alzheimer’s disease, not only preserves long-term potentiation but also mitigates the progression toward severe synaptic dysfunction.

Additionally, we evaluated the effects of Avn-C after treatment cessation by allowing a one-week (7-day) washout period following the three-month oral administration initiated at the early stage of disease in both AD mouse models. Compared to wild-type (WT) mice, both 5xFAD and Tg2576 mice showed a significant failure in the induction and maintenance of long-term potentiation (LTP) after treatment cessation (WT: 145% ± 0.6%; 5xFAD: 128% ± 1%; Tg2576: 129% ± 2%; *p* < 0.001, *n* = 3). No significant differences were observed between the two AD models post-treatment (Supplementary Figure S1a,b).

### 3.2. Sustained Avn-C Administration from Early AD Stage Preserves AMPK Activation, Inhibits GSK3β and Caspase-3, and Prevents Neurodegeneration

Building on our electrophysiological findings, we further investigated the molecular mechanisms underlying these effects. Our earlier research shows that Avn-C significantly enhances memory and cognitive function by binding to α1A adrenergic receptors, leading to AMPK activation, GSK3β inhibition, and reduced caspase–3–mediated neuronal cell death [22]. We employed the Western blot technique to obtain precise and reliable results in investigating critical protein expressions, focusing on the three-month administration of Avn-C, starting from the early stages of AD. Our studies revealed that AMPK activation requires phosphorylation at threonine 172 [28]. After three months of Avn-C administration from the early AD stage, results confirmed sustained AMPK activation, indicated by increased phosphorylation at the Thr-172 site. Specifically, compared to their respective vehicles, pAMPK levels were elevated by approximately ~45% in 5xFAD and ~37% in Tg2576 mice treated with Avn-C. In contrast, both vehicle AD models showed markedly reduced pAMPK levels during disease progression (*p* < 0.001 for 5xFAD; *p* < 0.05 for Tg2576; *n* = 4), based on hippocampal protein extracts. In line with our previous findings, several studies have demonstrated that AMPK activation inhibits GSK3β activity through phosphorylation at the serine 9 (Ser9) residue [29,30]. Hippocampal protein extracts from 5xFAD mice following long-term (three-month) oral Avn-C administration, initiated at the early AD stage, showed a marked ~27.3% increase in GSK3β Ser9 phosphorylation compared to 5xFAD vehicles (*p* < 0.001). Similarly, Tg2576 mice exhibited a significant ~66.8% increase in Ser9 phosphorylation relative to their vehicles (*p* < 0.01; *n* = 3). These findings indicate that Avn-C effectively inhibits GSK3β activity and maintains it throughout disease progression (Figure 1e).

Additionally, synaptic impairment during AD progression is closely linked to ongoing synaptic degeneration and neuronal loss mediated by apoptotic caspase-3 activation. Studies have shown that AD patients exhibit elevated levels of active caspase-3 (cleaved caspase-3, Clv.C-3), particularly enriched in the postsynaptic density (PSD), indicating its critical role in synaptic dysfunction and degeneration [31]. Analysis of hippocampal protein lysates from both vehicle AD mouse models revealed a substantial ~70–80% increase in cleaved caspase-3 (Clv.C-3) levels, indicating heightened neurodegeneration during disease progression (5xFAD vehicle: *p* < 0.001; Tg2576 vehicle: *p* < 0.01, vs. WT). However, long-term (three-month) oral Avn-C administration initiated at the early disease stage significantly reduced Clv.C-3 levels by approximately ~65% in both 5xFAD and Tg2576 mice compared to their respective vehicles (5xFAD Avn-C: *p* < 0.001; Tg2576 Avn-C: *p* < 0.01). Notably, Clv.C-3 levels in Avn-C-treated 5xFAD mice remained significantly higher than in WT (*p* < 0.05), while Tg2576 mice treated with Avn-C showed no significant difference from WT (*p* > 0.05), suggesting a more complete restoration in the Tg2576 model. These findings strongly support that early and sustained Avn-C treatment effectively reduces and stabilizes cleaved caspase-3 levels (ANOVA, *p* < 0.001, *n* = 3), thereby preventing excessive neuronal apoptosis and preserving synaptic integrity. (Figure 1f).

### 3.3. Three-Month Oral Administration of Avn-C from the Early AD Stage Attenuates Amyloidogenic Processing and Aβ1-42 Production

Amyloidogenic processing produces Aβ peptides (Aβ1-40 and Aβ1-42) from the transmembrane amyloid precursor protein (APP) through sequential cleavage by β-secretase (BACE1) followed by γ-secretase [32,33]. Studies have shown that AMPK activation during the early stages of Alzheimer’s disease can significantly reduce Aβ production. This reduction is achieved by promoting autophagic degradation of BACE1, the key enzyme responsible for generating Aβ from amyloid precursor proteins (APP) [34]. Due to limited efficacy, many drugs and BACE1 inhibitors have failed to effectively reduce Aβ production. Even in cases where BACE1 inhibitors successfully lowered Aβ levels, they often did not translate into cognitive improvement, highlighting the challenge of targeting Aβ alone for therapeutic benefit [35,36]. Our current study found that disease progression in vehicle 5xFAD mice led to a substantial increase in amyloidogenic markers, with APP levels elevated by ~68.1% and BACE1 by ~55.1% compared to wild-type vehicles (*p* < 0.001). In contrast, 5xFAD mice treated with Avn-C from the early disease stage showed only moderate elevations ~35.9% in APP (*p* < 0.05) and ~24.4% in BACE1 (*p* < 0.01) wild-type vehicles. Avn-C treatment significantly reduced APP and BACE1 levels by ~50.2% and ~40.4%, respectively (*p* < 0.001), compared to untreated 5xFAD mice. These findings suggest that early and sustained Avn-C administration effectively suppresses amyloidogenic processing, thereby potentially preventing Aβ-mediated AD progression.

**Figure 2:**
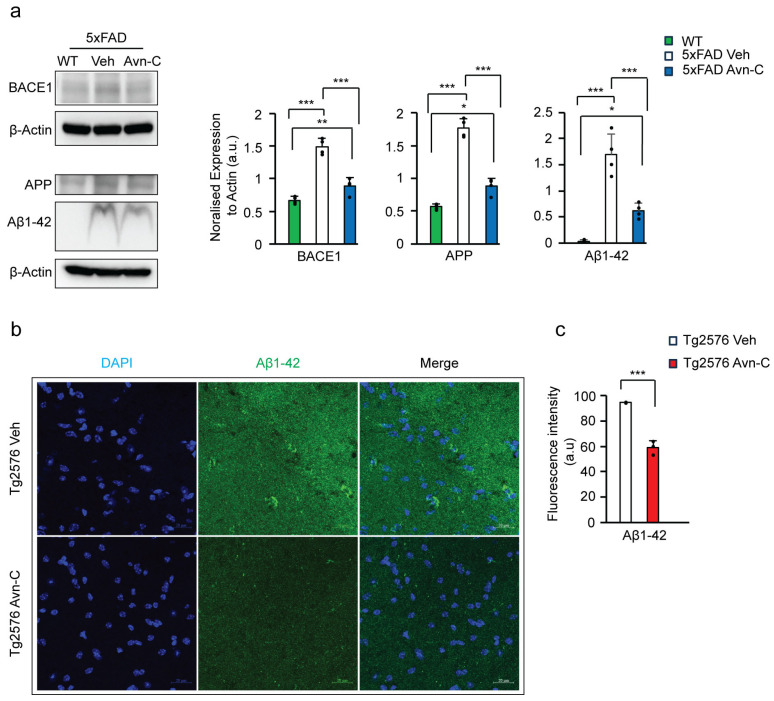
Long-term Avn-C oral administration initiated at the early AD stage lowers Aβ1-42 production and prevents amyloid-mediated disease advancement in AD mouse models. (**a**) Western blot and quantitative analysis of BACE1, APP, and Aβ1-42 levels from the hippocampal lysate of WT, 5xFAD (vehicle and Avn-C group) mice (*n* = 4). A severe amyloid-mediated disease progression was observed from the vehicle 5xFAD group with increased APP, BACE1, and Aβ1-42 levels compared to the WT mice, and Avn-C lowers these protein expressions and halts amyloid progression in 5xFAD mice orally administered with Avn-C three months from early AD stage. (**b**) Fluorescent confocal z-stack image of Aβ1-42 (green), DAPI (blue) from Tg2576 vehicle and 3 months Avn-C treated mice hippocampal sections (2 sections/group) focused on CA3 region (scale bar 20 µm). (**c**) Quantitative analysis of Aβ42 fluorescent intensities are shown in the graph (*n* = 3), where Aβ1-42 levels at the CA3 hippocampal region were lowered by the Avn-C administered early at AD stage with three months of continuous treatment. In all panels, the error bar indicates S.E.M. Overall differences among groups were analyzed using the unpaired *t*-test two-tailed, one-way ANOVA (*** *p* < 0.001), followed by pairwise comparisons between groups using post hoc Tukey’s test. Statistical significance is indicated as *** *p* < 0.001, ** *p* < 0.01, * *p* < 0.05.

The Aβ downregulation was also observed in other research where chronic neoline administration downregulated BACE1, and active AMPK influenced Aβ1-42 decrease [37]. To further assess amyloid pathology, we measured Aβ1-42 levels in hippocampal protein lysates from WT, vehicle 5xFAD, and Avn-C-treated 5xFAD mice. As expected, Aβ1-42 was undetectable or present at extremely low levels in WT mice (*p* < 0.001 vs. vehicle 5xFAD; *p* < 0.05 vs. 5xFAD Avn-C). In contrast, vehicle 5xFAD mice showed a dramatic ~97.8% increase in Aβ1-42 levels compared to WT, reflecting robust amyloidogenic activity. Remarkably, long-term Avn-C administration initiated at the early disease stage significantly reduced Aβ1-42 levels by ~63.6% compared to untreated 5xFAD mice (*p* < 0.001). This further supports Avn-C’s potential in mitigating Aβ-mediated AD progression (ANOVA, *p* < 0.001, *n* = 4) (Figure 2a).

Additionally, the level of Aβ1-42 was tested in the CA3 region of the hippocampal tissue sections from Tg2576 vehicle and Tg2576 3 months Avn-C. By analyzing the fluorescent intensity of Aβ1-42, the longer administered Avn-C initiated from early AD stage mice shows a ~37.7% reduction compared to the vehicle mice (*p* < 0.01, *n* = 3) (Figure 2b,c). Taken together, administering Avn-C from the early stage of the disease and sustaining the treatment for the long term is crucial to maintaining the synaptic potentiation alongside downregulating the amyloidogenic processing, which slows or prevents severe impairment in AD mouse models.

### 3.4. Long-Term Oral Administration of Avn-C from the Early AD Stage Attenuates GSK3β-Mediated Tau Hyperphosphorylation

**Figure 3:**
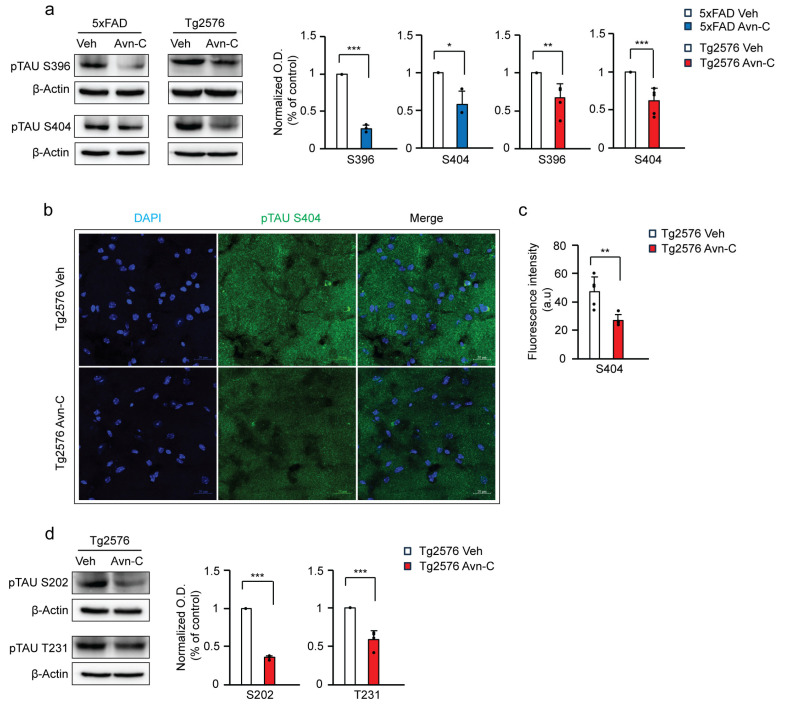
Three months of Avn-C administration starting from the early stages of the disease prevents tau hyperphosphorylation and the advancement of tau-mediated AD. (**a**) Western blots from hippocampal lysate tau hyperphosphorylation levels between vehicle and Avn-C oral administered AD mouse models, 5xFAD (Ser396 *n* = 4), (Ser404 *n* = 3), and Tg2576 (Ser396, 404 *n* = 5), (**b**) Fluorescent image confocal z-stack of hyperphosphorylated tau Ser404 (green) levels between vehicle and Avn-C-treated Tg2576 mice CA3 region hippocampal sections: two sections/mouse (*n* = 5) (scale bar 20 µm) (left). (**c**) Fluorescent quantitative analysis of Ser404 between vehicle and Avn-C Tg2576 mice and (**d**) Western blot representative band image with quantitative phosphorylation levels of (Ser202 *n* = 3), (Thr231 *n* = 4) revealed the Avn-C administration from early disease stage and that maintaining the treatment for longer duration lowers tau hyperphosphorylation and prevents further tau-mediated disease progression. Mean ± S.E.M.s, differences were considered significant, unpaired *t*-test two-tailed. Statistical significance indicated as *** *p* < 0.001, ** *p* < 0.01, * *p* < 0.05.

Tau hyperphosphorylation is another key feature of AD progression. While drugs like donepezil and rivastigmine reduce amyloid pathology in 5xFAD mice, they do not affect tau pathology [38], showing the complex independent mechanisms of tau disease pathology, leading to severe cognitive impairment. As amyloid-β directly activates GSK3β, driving tau hyperphosphorylation [39,40], we assessed GSK3β-mediated tau hyperphosphorylation at serine/threonine sites via Western blot using hippocampal protein extracts from AD mice to evaluate the impact of long-term Avn-C treatment, initiated at the early AD stage, on preventing tau pathology–driven disease progression. Long-term Avn-C administration, initiated at the early AD stage, significantly reduced tau hyperphosphorylation in 5xFAD and Tg2576 mice. In 5xFAD mice, phosphorylated tau levels decreased by ~73.9% at Ser396 (*p* < 0.001, *n* = 4) and ~42.2% at Ser404 (*p* < 0.05, *n* = 3). Similarly, Tg2576 mice showed a ~33% reduction at Ser396 (*p* < 0.01, *n* = 5) and ~38.6% at Ser404 (*p* < 0.001, *n* = 5) compared to their respective vehicles (Figure 3a).

Moreover, immunostaining of hippocampal sections further confirmed these findings. Quantitative analysis revealed a ~46.3% reduction in tau Ser404 deposition in the CA3 region of Avn–C-treated Tg2576 mice versus vehicles (*p* < 0.01, *n* = 5), indicating that Avn-C effectively limits tau pathology when administered from the early stages of AD (Figure 3b,c). Additionally, in Tg2576 mice, Avn-C treatment significantly reduced tau hyperphosphorylation by ~64.7% at Ser202 (*n* = 3) and ~41.1% at Thr231 (*n* = 4) compared to vehicles (*p* < 0.001; Figure 3d). These findings further support Avn-C’s disease-modifying potential, particularly when sustaining administration from the early stages of AD, by effectively preventing tau hyperphosphorylation in the hippocampus of AD mouse models.

### 3.5. Avn-C Administration from the Early AD Stage Sustains Anti-Inflammatory Effects and Prevents Neuroinflammation

NF-κB is an upstream regulator of proinflammatory cytokine production. To determine whether sustained Avn-C administration maintains its anti-inflammatory effects, we analyzed the expression of phosphorylated IKKα/β (pIKKα/β Ser176/180)—a central mediator of the canonical NF-κB pathway [41]. Western blot was performed to assess whether three months of Avn-C treatment from the early AD stage could help halt or slow neuroinflammation in AD mice. During AD progression, vehicle 5xFAD mice showed a significant ~72% increase in pIKKα/β levels compared to WT (*p* < 0.01). However, three months of Avn-C treatment, initiated at the early AD stage, reduced pIKKα/β levels by ~62% compared to vehicle 5xFAD mice (*p* < 0.05).

**Figure 4:**
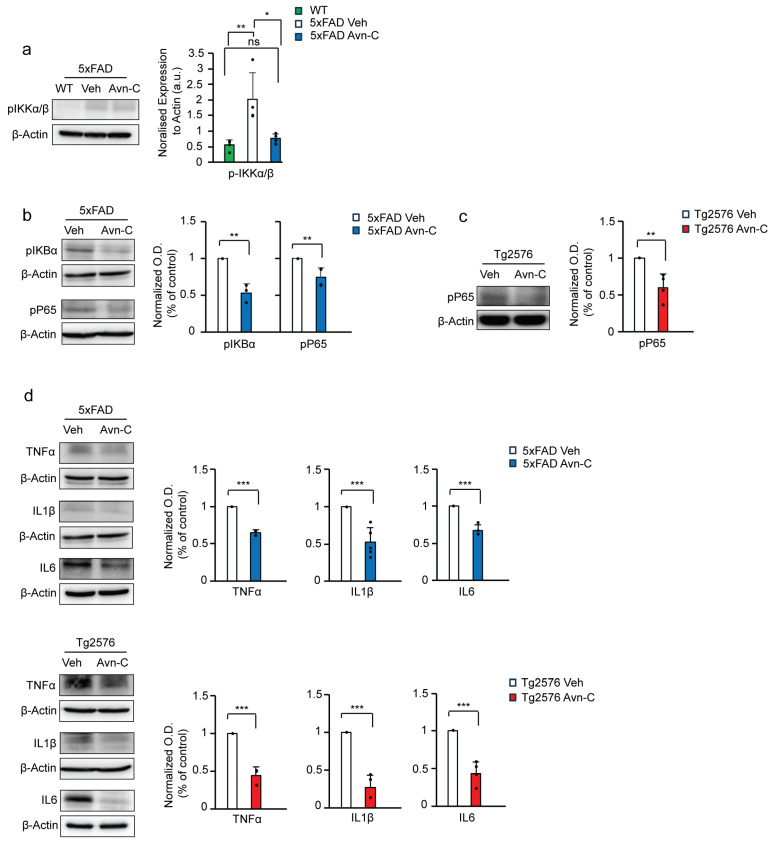
Three months of Avn-C oral administration from the early AD stage attenuates NF-kB activation and proinflammatory cytokine production during the AD progression. (**a**) Western blot and quantitative analysis of active p-IKK⍺/β levels between WT, vehicle, and long-term Avn-C-treated 5xFAD mice from hippocampal protein extract (*n* = 4). (**b**,**c**) Western blot and quantitative analysis from the hippocampal lysate of AD mouse models, vehicle, and 3 months Avn-C-administered 5xFAD mice active p-IKB⍺ (*n* = 3), p-p65 (*n* = 4) and Tg2576 (vehicle and 3 months Avn-C) active p-p65 (*n* = 4) protein expression levels demonstrated that Avn-C initiated at the early AD stage and continued for 3 months significantly reduces and halts the NF-kB signaling from activation. (**d**) Proinflammatory cytokines from 5xFAD (vehicle and 3 months Avn-C) TNF⍺ (*n* = 3), IL6 (*n* = 4), IL1β (*n* = 5) and Tg2576 (vehicle and 3 months Avn-C) TNF⍺ (*n* = 3), IL6, IL1β (*n* = 4) levels of hippocampal protein extract Western blot and quantitative analysis demonstrating that Avn-C preserved its anti-inflammatory properties and maintained the lowered proinflammatory cytokine production levels in both AD mouse models. Mean ± S.E.M.s. Overall differences among groups were analyzed using unpaired *t*-test two-tailed, one-way ANOVA (** *p* < 0.01) followed by pairwise comparisons between groups performed using post hoc Tukey’s test. Statistical significance is indicated as *** *p* < 0.001, ** *p* < 0.01, * *p* < 0.05, and ^ns^ *p* > 0.05 (no significance).

Notably, there was no significant difference between WT and Avn-C-treated 5xFAD mice (*p* > 0.05), indicating that long-term Avn-C administration effectively suppresses IKKα/β activation, restoring it to baseline levels. Collectively, the longer Avn-C treatment from the early disease stage prevents IKK complex activation by lowering the phosphorylation level of IKK⍺/β in 5xFAD AD mice (ANOVA, *p* < 0.01, *n* = 4) (Figure 4a).

IKBα acts as an inhibitor of NF-κB. Upon phosphorylation by active pIKKα/β, IKBα is detached from NF-κB, leading to its degradation via ubiquitination. This releases the active NF-κB subunit pP65, which translocates into the nucleus and promotes the transcription of proinflammatory cytokines [42,43,44,45]. Long-term Avn-C treatment initiated at the early AD stage significantly reduced the phosphorylation of IκBα (~46.8%, *p* < 0.01, *n* = 3), thereby limiting NF-κB activation in 5xFAD mice. Correspondingly, levels of phosphorylated NF-κB p65 (Ser536) were reduced by ~25.1% in Avn–C-treated 5xFAD mice compared to vehicles (*p* < 0.01, *n* = 4). Similarly, Tg2576 mice treated with Avn-C for three months exhibited a ~40.3% decrease in p-p65 levels versus vehicle Tg2576 mice (*p* < 0.01, *n* = 4). These results indicate that Avn-C effectively suppresses NF-κB signaling and associated neuroinflammatory responses (Figure 4b,c). We further analyzed the mature forms of proinflammatory cytokines in hippocampal tissue lysates. In 5xFAD mice, sustained Avn-C treatment initiated at the early AD stage significantly reduced cytokine levels by ~35.3% for TNFα (*n* = 3), ~32.3% for IL-6 (*n* = 4), and ~47.8% for IL-1β (*n* = 5) compared to vehicle 5xFAD mice (*p* < 0.001). Notably, Tg2576 mice treated with Avn-C for three months exhibited an even greater reduction ~55.7% for TNFα (*n* = 3), ~55.9% for IL-6 (*n* = 4), and ~73.1% for IL-1β (*n* = 4) versus vehicle Tg2576 mice (*p* < 0.001), demonstrating potent and sustained anti-inflammatory effects (Figure 4d). In a compelling comparison, Tg2576 AD mice treated with Avn-C exhibited a significantly greater and more sustained reduction in proinflammatory cytokines than Avn-C-treated 5xFAD mice. This notable difference underscores the distinct pharmacokinetic response to Avn-C between the models, highlighting its capacity to provide enduring anti-inflammatory effects when administered from the early stages of Alzheimer’s disease.

### 3.6. Long-Term Avn-C Treatment from the Early AD Stage Modulates Microglial Morphology and Reduces Chronic Activation

**Figure 5:**
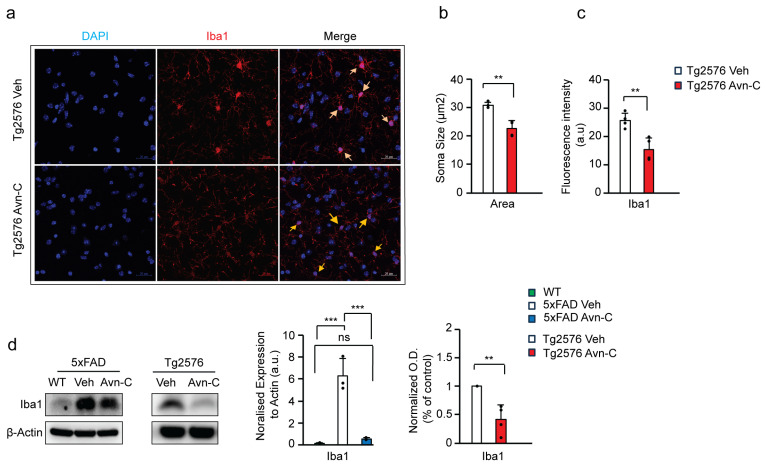
Early AD-stage Avn-C administration with long-term treatment prevents chronic activation and reduces the cell body (soma) size of microglial cells in AD mouse models. (**a**–**c**) The confocal z-stack fluorescent image Iba1 (red) microglial marker and DAPI (blue) nuclei. Quantitively, cell area (soma, yellow arrow) and fluorescent intensity are measured from 5xFAD vehicle and 3 months Avn-C orally administered mice hippocampal sections (2 sections/mouse) (*n* = 4); for cell soma size from hippocampal sections, 6–7 cells/sections were analyzed (scale bar 20 µm). (**d**) Western blot and quantitative analysis of IBA1 microglial marker from WT, vehicle, and 3 months Avn-C-treated (5xFAD (*n* = 3) and Tg2576 (*n* = 4)) mice hippocampal lysate. Mean ± S.E.M.s. Overall differences among groups were analyzed using unpaired *t*-test two-tailed, one-way ANOVA (*** *p* < 0.001), and pairwise comparisons between groups were performed using post hoc Tukey’s test. Statistical significance is indicated as *** *p* < 0.001, ** *p* < 0.01, ^ns^ *p* > 0.05 (no significance).

Iba1 (Ionized calcium-binding adaptor molecule 1) is a microglial cell marker. In Alzheimer’s disease, elevated Iba1 expression reflects chronic microglial activation, often driven by increased Aβ levels. While not inherently beneficial, this sustained activation alters microglial morphology, characterized by enlarged cell bodies, and promotes proinflammatory cytokine production, ultimately accelerating disease progression [46,47,48,49,50]. To evaluate the impact of long-term Avn-C treatment initiated at the early AD stage on microglial activation and morphology, we measured the area of the microglial cell body (soma), marked by Iba1, in the CA3 region of the hippocampus in Tg2576 mice. This analysis helps determine whether Avn-C can modulate chronic microglial reactivity associated with AD progression. Compared to vehicle Tg2576 mice, long-term Avn-C treatment initiated at the early AD stage significantly reduced microglial soma size by ~26.5% (*p* < 0.01, *n* = 4), indicating a shift away from chronic activation. As Iba1 is a well-established marker of microglial activation [51,52], we quantified its expression via fluorescent intensity in the CA3 region of hippocampal tissue from Tg2576 mice (vehicle vs. Avn-C treated). The analysis revealed a significant ~39.4% reduction in Iba1 fluorescent intensity in the Avn-C treated group compared to vehicles (*p* < 0.01, *n* = 4) (Figure 5a–c). This reduction further suggests that Avn-C effectively attenuates microglial reactivity in the Tg2576 AD model. Western blot analysis of Iba1 protein expression from hippocampal tissue lysates revealed that 5xFAD vehicle mice at advanced AD stages exhibited a ~97.3% increase in Iba1 levels compared to WT mice (*p* < 0.001).

In contrast, long-term Avn-C treatment initiated from the early AD stage in 5xFAD mice showed significantly suppressed Iba1 expression by ~90% relative to 5xFAD vehicles (*p* < 0.001), with no significant difference between WT and Avn-C-treated 5xFAD mice (*p* > 0.05). These results indicate that Avn-C effectively prevents chronic microglial activation in 5xFAD mice (ANOVA, *p* < 0.001, *n* = 3). Similarly, Avn-C treatment in Tg2576 mice led to a ~58.6% reduction in Iba1 expression, indicating attenuated microglial activation (*p* < 0.01 vs. Tg2576 vehicle) (Figure 5d). These findings demonstrate that Avn-C modulates microglial cells at the cellular level by preventing Aβ-induced chronic microglial activation. This modulation is consistent with the observed reduction in proinflammatory cytokine production in the hippocampus of AD mice, further supporting Avn-C’s long-term potential for neuroprotective and anti-inflammatory effects.

### 3.7. Early and Sustained Avn-C Treatment Reduces Large Amyloid Plaques and Promotes Microglial Barrier Formation Around Smaller Plaques in the Hippocampus

Understanding microglia–amyloid plaque interactions is key to developing therapies that target plaque-related damage [53,54]. Moreover, microglia with high TREM2 expression cluster around early Aβ plaques, helping insulate and limit their spread [55]. Although long-term Avn-C treatment from the early AD stage significantly reduces Aβ1-42 levels, evidence for plaque clearance in 5xFAD mice was initially unclear.

**Figure 6:**
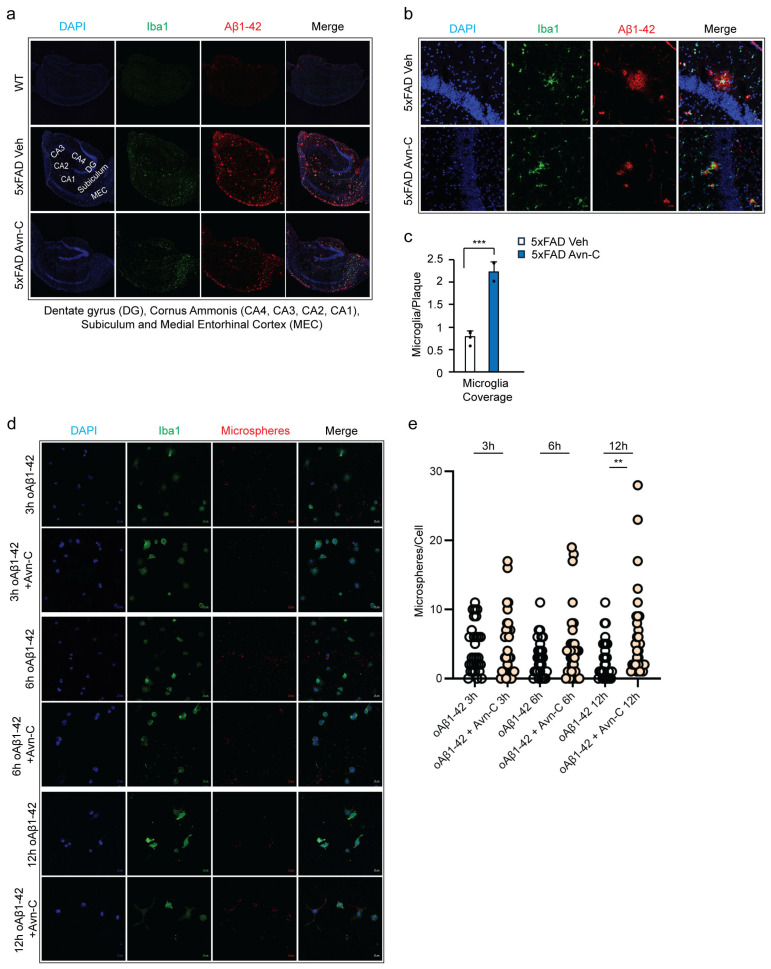
Long-term oral administration of Avn-C from the early AD stage reduces large amyloid plaque deposition, strengthens the microglial cell barrier in hippocampal tissue, and protects and enhances microglial phagocytosis in BV2 cells. (**a**) The representative confocal z-stack fluorescent image DAPI (blue), Iba1 (green), and Aβ1-42 (red) focused on microglial recruitment and plaque deposition in the hippocampus proper and medial entorhinal cortex between vehicle and 3 months Avn-C oral administered hippocampal tissue sections (scale bar 200 µm). (**b**) Fluorescent confocal z-stack image from 5xFAD vehicle and three months Avn-C-administered mice hippocampal sections (50 µm, two sections per mouse) fluorescent tag with Iba1 (green) and Aβ42 (red) (scale bar 20 µm). (**c**) Quantification of the area covered by microglia over the plaque between vehicle and three months Avn-C orally administered mice from early AD stage from hippocampus proper. The ratio is calculated as (area of microglia)/(area of plaque) in the region of interest. Number of plaque analyzed 30/group (*n* = 4). (**d**) Confocal fluorescent image of DAPI (blue) and BV-2 microglial cells (green) pre-activated by oAβ1-42 (1.0 µM) for 30 min and continued treatment with oAβ1-42 (1.0 µM) and oAβ1-42 (1.0 µM) + Avn-C (50 µm) for different time points: 3, 6, and 12 h. Phagocytosis was observed from ingested fluorescent carboxylate microsphere (red) (scale bar 20 µm). (**e**) Quantification of phagocytosis from BV-2 cells treated with oAβ1-42 (1.0 µM) or oAβ1-42 (1.0 µM) + Avn-C (50 µm), analyzed based on the number of microspheres engulfed per cells from 3, 6, and 12 h time duration; 30 cells analyzed per condition. Mean ± S.E.M.s. Differences were analyzed using an unpaired *t*-test, two-tailed. Statistical significance is indicated as *** *p* < 0.001, ** *p* < 0.01.

To investigate this, we immunostained hippocampal brain sections from WT, vehicle 5xFAD, and Avn-C-treated 5xFAD mice using Iba1 and Aβ1-42. The results showed that vehicle 5xFAD mice had extensive large and small plaque deposits across the hippocampal subregions (CA4-CA1) and the medial entorhinal cortex (MEC), indicating advanced plaque accumulation during disease progression. In contrast, hippocampal sections from 5xFAD mice treated long-term with Avn-C from the early AD stage showed a marked reduction in large amyloid plaque accumulation within the hippocampus proper (Figure 6a). This reduction may stem from decreased Aβ1-42 production and enhanced microglial phagocytic activity. To further examine microglial interaction with plaques, we quantified the coverage area of microglial cells associated with plaques (30 plaques/mouse). In hippocampal sections of long-term Avn-C-treated 5xFAD mice, particularly within the hippocampus proper, microglial coverage around plaques increased significantly, by ~64.8% (*p* < 0.001, compared to vehicle 5xFAD, *n* = 4). This enhanced envelopment formed a protective barrier around small plaques, potentially limiting their spread and offering neuroprotection (Figure 6b,c). These findings and the observed reduction in Aβ1-42 production demonstrate that long-term Avn-C treatment initiated at the early AD stage effectively reduces plaque accumulation in the hippocampus of 5xFAD mice, particularly within the CA4–CA1 regions. This reduction likely underlies the restored synaptic function observed in electrophysiological recordings.

### 3.8. Avn-C Protects Microglial Cells from Oligo-Aβ1-42-Induced Phagocytic Impairment

Based on these observations, we next assessed whether Avn-C directly enhances phagocytic activity. In vitro experiments using BV2 microglial cells revealed that treatment with 1.0 µM oligomeric Aβ (oAβ) progressively impaired phagocytosis over 3–12 h, while exposure to 10 µM oAβ significantly reduced cell viability [56].

To determine whether Avn-C can counteract oAβ-induced impairment of microglial phagocytosis, BV2 microglial cells were pre-activated with 1.0 µM oAβ1-42 for 30 min in both vehicle and treatment groups.

Following activation, the Avn-C group received 50 µM Avn-C and was incubated for 3, 6, or 12 h. At each time point, both vehicle and Avn-C-treated cells were exposed to crimson-fluorescent carboxylate-modified microspheres (1 µm diameter, 50 microspheres/cell) for 30 min to assess phagocytic uptake (Figure 6d). The phagocytosis was assessed in BV2 microglial cells treated with oAβ1-42 alone or combined with Avn-C across 3, 6, and 12 h time points (30 cells/group). In the oAβ1-42-only group, an initial transient phagocytic response was observed at 3 h, followed by a ~30% decline at 6 h and a further ~7% decrease by 12 h. In contrast, the oAβ1-42 + Avn-C group maintained a stable phagocytic response between 3 h and 6 h (with less than ~1% increase, insignificant). Notably, at 12 h, Avn-C treatment significantly enhanced phagocytosis by ~25% compared to the 6 h time point (*p* < 0.01), indicating Avn-C’s potential in safeguarding the cells from toxic oAβ1-42 and restoring microglial phagocytic function over time (Figure 6e).

## 4. Discussion

Preventing or delaying the progression may not represent the ultimate solution for AD. However, it is essential to recognize that the priorities must shift in the absence of a cure. Restoring and maintaining impaired synaptic potential while protecting neurons from irreversible damage for as long as possible are worthy goals for new drug therapeutic interventions to preserve memory in AD. Achieving these goals is most effective when treatment is initiated early in the disease’s development stage. However, studies showed that many drugs only provided short-term benefits at the early disease stage, and over time, as the disease continued to progress, the drug lost its effectiveness and failed to prevent AD progression [57,58]. This study using AD mouse models provides strong evidence for the therapeutic potential of long-term Avn-C treatment, particularly when administered orally from the early stages of the disease. Our research focused on the hippocampus, a brain region critical for learning and memory, which is especially vulnerable during AD progression and closely linked to the onset of cognitive decline and memory loss [59]. Results from hippocampal slice electrophysiology (fEPSP recordings) confirmed that early-stage sustained Avn-C administration is critical for rescuing impaired LTP and maintaining it in AD mouse models. However, a 7-day post-treatment cessation led to the loss of the synaptic plasticity previously restored by Avn-C, suggesting that the aggressive pathology in 5xFAD and Tg2576 mice rapidly overcomes therapeutic gains in the absence of continued treatment. These findings underscore the importance of early and sustained Avn-C administration to effectively counteract disease progression. Moreover, continued treatment preserves this recovery, with Avn-C maintaining normal synaptic activity and preventing further synaptic dysfunction. Synaptic activity depends on stable cellular energy, but early Aβ oligomer buildup disrupts energy metabolism and impairs AMPK function [60]. Initiating Avn-C administration from the early AD stage and continuing for the long term preserves AMPK activation and overcomes the inhibition caused by Aβ, confirming that Avn-C regulates the initiation and maintenance of LTP by ensuring a reliable energy supply for synapses. Apart from restoring and maintaining the synaptic function from progressive impairment, the therapeutic drug must effectively protect the brain from severe neurodegeneration. The brains of human AD patients contain a high level of Aβ and active caspase-3 [61], which promotes apoptosis and causes severe irreversible memory loss. Sustained Avn-C treatment from the early AD stage keeps cleaved caspase-3 levels low, reducing apoptosis, early synaptic degeneration, and neuronal loss in the hippocampal tissue. Additionally, GSK3β, a key regulator in AD pathology, is hyperactivated, contributing to amyloid accumulation, tau hyperphosphorylation, neuroinflammation, and synaptic dysfunction, including impaired LTP [62].

Major drug development efforts primarily focus on inactivating this critical kinase [63,64]. Previous studies on 14 days of Avn-C treatment from the early stage showed significant inhibition of GSK3β. In this study, we demonstrate that sustaining this treatment in the long term allows Avn-C to maintain its inhibitory effect on abnormal GSK3β activation, effectively regulating disease pathology and preventing disease progression in AD mouse models. Several natural compounds showed anti-amyloid and anti-tau effects. With low adverse effects, these compounds have a distinct advantage over other synthetic compounds. This reduces the amyloid and tau aggregation by inhibiting secretase or preventing secondary structure modification. However, these compounds lack evidence on in vivo tests and the durability of their impact when the disease advances is not well documented [65,66]. Our present study on Avn-C demonstrated both anti-amyloid and anti-tau properties by lowering the amyloidogenic processing proteins BACE1 and APP, which reduces the Aβ1-42 production and prevents tau hyperphosphorylation at Ser 202, 396, 404, and Thr 231 sites in the hippocampal tissue, effectively preventing amyloid- and tau-mediated disease advancement in AD mouse models. During AD progression, active caspase-3 alongside BACE1 also cleaves APP, contributing to Aβ production and synaptic loss [67]. Our findings suggest that early Avn-C treatment inhibits caspase-3 and GSK3β activation while stimulating AMPK. With long-term administration, these effects reduce Aβ1-42 production, plaque deposition, and tau hyperphosphorylation, ultimately slowing AD progression in model mice. This highlights Avn-C as a promising therapeutic candidate capable of restoring and sustaining LTP while mitigating Aβ and tau pathologies.

Multiple studies have reported elevated TNF⍺, IL6, IL1β, and hyperactive microglia in AD patients with early mild cognitive impairment (MCI) [68], contributing to LTP impairment via Aβ-induced proinflammatory cytokines [69]. Studies have shown that Avenanthramides, including Avn-C, exhibit anti-inflammatory properties [70]. The early Avn-C treatment reduces cytokine production by inhibiting the NF-κB pathway [22]. Long-term oral administration further suppresses proinflammatory cytokine production, effectively halting neuroinflammation-driven disease progression in the hippocampus of AD mouse models. Additionally, chronic microglial activation by accumulating Aβ oligomers elevates proinflammatory cytokine levels, creating a toxic environment that leads to neuronal damage as AD progresses [71]. Interestingly, when early and sustained Avn-C treatment limits excessive amyloid and tau pathology, analysis with the microglial-specific marker Iba1 shows that Avn-C prevents chronic microglial activation and preserves their long-term neuroprotective function. This restoration of normal microglial activity supports the formation of a protective barrier around smaller amyloid plaques, effectively restricting their spread in the hippocampus of AD mice. Notably, this microglia-mediated barrier function has also been reported in other studies, where microglial cells are found to be significantly lowered when the plaque size increases during AD progression [72,73].

**Figure 7:**
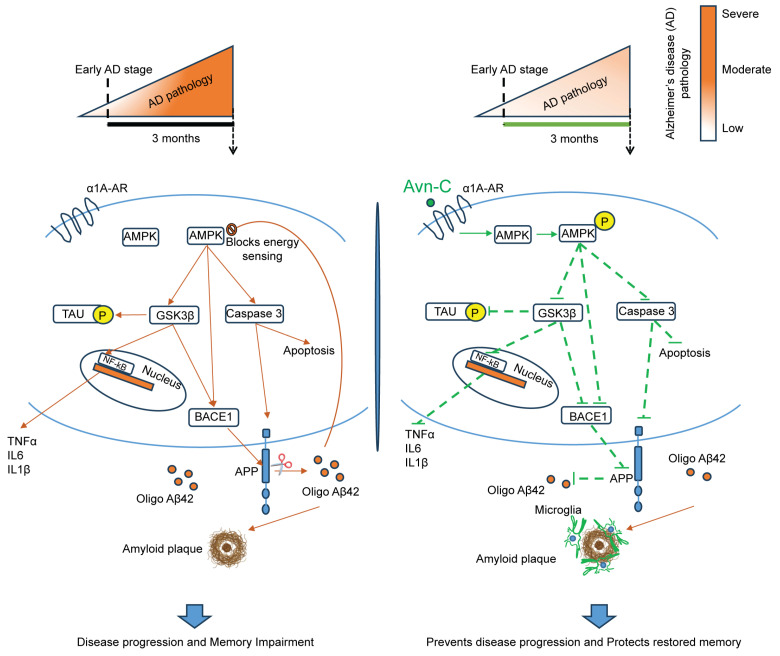
Overall schematic representation of long-term (three months) Avn-C administration initiated from early AD stage restores and maintains synaptic plasticity and prevents AD progression. Early Avn-C administration is essential in managing Alzheimer’s disease effectively; with sustained long-term treatment, Avn-C can significantly delay the onset of more severe cognitive impairment. During disease progression, the overproduction of Aβ impairs the AMPK signaling and activates the GSK3β, which negatively modulates multiple signaling pathways, including the hyperphosphorylation of tau, neuroinflammation, and amyloid plaque deposition, which highly promotes neurodegeneration during disease development and leads to severe cognitive impairment. The long-term (three months) Avn-C administration from the early disease stage significantly preserves the synaptic potentiation and memory; it maintains its beneficial properties by sustaining the activation of AMPK and inhibition of GSK3β, which further prevents the activation of NF-kB inflammatory mechanisms, thereby reducing proinflammatory cytokine production, and reduces the hyperphosphorylation of tau at multiple serine/threonine sites. Furthermore, the long-term treatment highly suppresses the caspase-mediated cell death and prevents the amyloidogenic processing, which reduces the amyloid plaque deposits and increases plaque clearance via brain resident microglial cells and prevents the disease development to more severe cognitive condition in the hippocampal tissue of AD mouse models.

Avn-C administration initiated at the early stage protects microglial cells, and long-term treatment prevents their chronic activation by disease pathology. This raises a key question: can Avn-C restore and enhance microglial phagocytosis to prevent abnormal aggregate accumulation during disease progression? To address this, in vitro studies using BV2 microglial cells showed that Avn-C protects against oAβ1-42-induced phagocytic impairment between 3 and 6 h, significantly improving phagocytosis at 12 h. These findings confirm Avn-C’s protective effect against Aβ-mediated dysfunction and its ability to restore microglial phagocytic function. However, the underlying molecular mechanisms driving this restoration require further investigation (Figure 7). These preclinical findings from AD mouse models highlight the promising potential of the natural compound Avenanthramide-C as an innovative early-stage AD treatment. Long-term Avn-C treatment not only significantly slows disease progression but also modulates key pathological processes, positioning it as a strong candidate for long-term efficacy in preventing neurodegeneration and advancing Alzheimer’s therapy.

## Data Availability

The original contributions presented in this study are included in the article/Supplementary Material. Further inquiries can be directed to the corresponding authors.

## Supplementary Materials

The following supporting information can be downloaded at: https://www.mdpi.com/article/10.3390/cells14110826/s1. Figure S1. One week post-treatment cessation after three months of oral administration of Avn-C from the early AD stage fails to induce/maintain LTP.

## Abbreviations

AD	Alzheimer’s disease
Avn-C	Avenanthramide-C
APP	Amyloid precursor protein
Aβ1-42	Amyloid-beta 1-42
oAβ1-42	Oligomer Amyloid beta1-42
fEPSP	Field excitatory postsynaptic potential
LTP	Long-term potentiation
CA	Cornu Ammonis
AMPK	Adenosine monophosphate-activated protein kinase
GSK3β	Glycogen synthase kinase three β
BACE1	Beta-site amyloid precursor protein cleaving enzyme 1
TNF⍺	Tumor necrosis factor-alpha
IL6	Interleukin 6
IL1β	Interleukin 1 beta
Iba1	Ionized calcium-binding adaptor molecule 1
