# Supplementary material for: Avenanthramide-C as Alzheimer’s Disease-Modifying Therapy: Early and Sustained Intervention Prevents Disease Progression in Mouse Models
Source: Cells. 2025 Jun 2;14(11):826. doi: 10.3390/cells14110826 (PMC12154218; doi:10.3390/cells14110826)
Supplement: Supplementary file 1 [file cells-14-00826-s001.zip › cells-3654251- Supplementary Figure.pdf]

Supplementary Figure

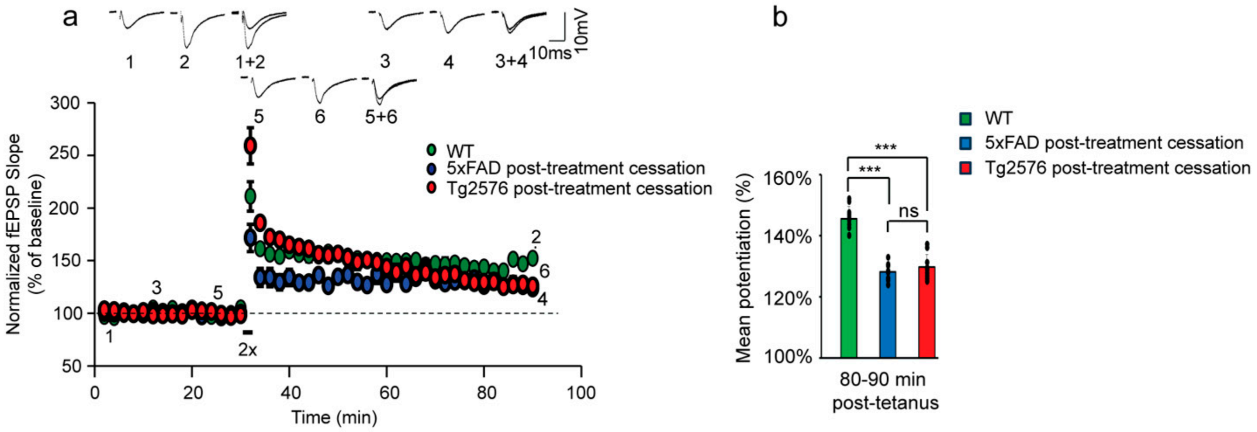

Supplementary Figure S1: One week post-treatment cessation after three months of oral administration of Avn-C from the early AD stage fails to induce/maintain LTP. a) fEPSP recording from WT, 5xFAD and Tg2576 (post-treatment cessation for 7 days) AD mouse showing in both models 7 days cessation after three months Avn-C treatment fails the induction and maintenance of LTP compared to the WT mice, recorded for one hour after 30 min stable baseline maintenance from the CA3 region of the hippocampal slice. The LTP was evoked by the two trains (2x) of tetanus stimulation at 100Hz for 1 second. The black box symbol in LTP data indicates tetanus stimulation (2X). b) The level of mean potentiation of last 10 min (80-90 min) post-2x Tetanus shows the Showed transient effects of synaptic potential after cessation of Avn-C (WT - 145% ± 0.6%, 5xFAD post-treatment cessation - 128% ± 1%, Tg2576 post-treatment cessation - 129% ± 2%) (*n* = 3). Mean ± S.E.M.s, Overall differences among groups were analysed using One-way ANOVA (***p < 0.001) and pairwise comparisons between groups using *post-hoc* Tukey's test. Statistical significance is indicated as ***p < 0.001, **p < 0.01, *p < 0.05 and ^{ns}p > 0.05 (no significance).
